# Induction of Mitochondrial Fragmentation and Mitophagy after Neonatal Hypoxia–Ischemia

**DOI:** 10.3390/cells11071193

**Published:** 2022-04-01

**Authors:** Syam Nair, Anna-Lena Leverin, Eridan Rocha-Ferreira, Kristina S. Sobotka, Claire Thornton, Carina Mallard, Henrik Hagberg

**Affiliations:** 1 Centre of Perinatal Medicine and Health, The Sahlgrenska Academy, University of Gothenburg, 41685 Gothenburg, Sweden; anna-lena.leverin@neuro.gu.se (A.-L.L.); eridan.rocha.ferreira@gu.se (E.R.-F.); kssobotka1@gmail.com (K.S.S.); carina.mallard@neuro.gu.se (C.M.); henrik.hagberg@obgyn.gu.se (H.H.); 2 Institute of Neuroscience and Physiology, The Sahlgrenska Academy, University of Gothenburg, 41390 Gothenburg, Sweden; 3 Institute of Clinical Sciences, The Sahlgrenska Academy, University of Gothenburg, 41685 Gothenburg, Sweden; 4 Department of Comparative Biomedical Sciences, Royal Veterinary College, London NW1 0TU, UK; cthornton@rvc.ac.uk

**Keywords:** mitochondria, metabolism, mitochondrial fission, neonatal brain injury, reactive oxygen species, neonatal hypoxia–ischemia, mitophagy

## Abstract

Hypoxia–ischemia (HI) leads to immature brain injury mediated by mitochondrial stress. If damaged mitochondria cannot be repaired, mitochondrial permeabilization ensues, leading to cell death. Non-optimal turnover of mitochondria is critical as it affects short and long term structural and functional recovery and brain development. Therefore, disposal of deficient mitochondria via mitophagy and their replacement through biogenesis is needed. We utilized mt-Keima reporter mice to quantify mitochondrial morphology (fission, fusion) and mitophagy and their mechanisms in primary neurons after Oxygen Glucose Deprivation (OGD) and in brain sections after neonatal HI. Molecular mechanisms of PARK2-dependent and -independent pathways of mitophagy were investigated in vivo by PCR and Western blotting. Mitochondrial morphology and mitophagy were investigated using live cell microscopy. In primary neurons, we found a primary fission wave immediately after OGD with a significant increase in mitophagy followed by a secondary phase of fission at 24 h following recovery. Following HI, mitophagy was upregulated immediately after HI followed by a second wave at 7 days. Western blotting suggests that both PINK1/Parkin-dependent and -independent mechanisms, including NIX and FUNDC1, were upregulated immediately after HI, whereas a PINK1/Parkin mechanism predominated 7 days after HI. We hypothesize that excessive mitophagy in the early phase is a pathologic response which may contribute to secondary energy depletion, whereas secondary mitophagy may be involved in post-HI regeneration and repair.

## 1. Introduction

It is well established that mitochondrial dysfunction and oxidative stress contribute to cell death following neonatal hypoxia–ischemia (HI) injury [1,2,3]. Defective mitochondria can contribute to oxidative stress and bioenergetic deficits, compromising cellular function [4,5]. The cell removes damaged mitochondria by a specialized autophagic pathway called mitophagy, which mediates the lysosome-dependent clearance of damaged mitochondria, essential for mitochondrial quality control [4]. Exposure to HI leads to neonatal brain injury mediated by mitochondrial stress through the disturbance of Ca^2+^ regulation and marked ROS production [6]. Data from our laboratory and others strongly suggest that mitochondrial dysfunction acts as a hub for these diverse injury responses [7,8,9,10,11,12,13].

If damaged mitochondria cannot be repaired, mitochondrial permeabilization ensues, leading to cell death [14]. This can lead to non-optimal turnover of mitochondria, particularly critical in the immature brain, as it may affect not only short and long-term structural and functional recovery but also brain development [15,16]. Therefore, the disposal of deficient mitochondria via mitophagy and their replacement through mitochondrial biogenesis is needed [17]. However, data describing the role of mitophagy following neonatal HI are scarce; a single study reports that the deficit in elimination of damaged/dysfunctional mitochondria in the male brain following HI may contribute to male vulnerability to neuronal death and long-term neurobehavioral deficits following HI [18].

The main elaborated mechanisms of mitophagy include PTEN-induced kinase 1 (PINK1) and Parkin. They mediate a mitochondrial quality control pathway, which leads to the removal of damaged mitochondria by selective autophagy [19]. The availability of additional mitochondrial recycling pathways including the BNIP3, NIX and FUNDC1 underline the importance of mitophagy [20,21]. Once mitophagy is triggered, mitochondria-localized receptors bind to LC3B [22] and regulate mitophagy by targeting impaired mitochondria to autophagosomes [21,23].

In our current study, we investigated mitochondrial dynamics (fission, fusion, and mitochondrial autophagy (mitophagy) after HI in vivo in mice and oxygen/glucose deprivation (OGD) in vitro in primary neurons. We also analyzed genes/proteins related to mitophagy after HI including PINK1/Parkin, BNIP3/NIX and FUNDC1. Our data suggest that OGD triggers mitochondrial fragmentation and an early increase in mitophagy. Western blotting indicated that both PINK1/Parkin-dependent and -independent mechanisms were increased immediately after OGD. Thereafter, mitochondrial morphology normalizes, accompanied by a decrease in mitophagy. Measurement of mitophagy after HI in brain tissue showed an initial increase in mitophagy followed by a delayed phase of mitophagy, which occurs only in the injured ipsilateral hemisphere.

## 2. Materials and Methods

### 2.1. Animals of In Vitro Experiments

Pregnant C57BL/6 dams were sourced from Charles River Laboratories International (Sulzfeld, Germany). Animals were housed and bred at the University of Gothenburg’s Laboratory for Experimental Biomedicine under specific pathogen-free conditions on a 12 h light–dark cycle (illuminated 07:00–19:00) at constant temperature (24 °C) and relative humidity (50–60%) with ad libitum access to food and water. All experiments were approved by the local ethical committee at the University of Gothenburg (No: 203-2014 and 32-2016) and performed according to the Guidelines for the care and use of Laboratory Animals. The mt-Keima mouse line was purchased from Jackson Laboratory [24]. The mt-Keima mouse has a pH-dependent coral-derived protein Keima, which is targeted to the mitochondrial matrix with a mitochondria-targeting sequence from COX VIII, to allow for the detection of mitophagy [24] which is resistant to lysosomal degradation. The Keima protein changes color depending on the pH environment when being transferred from an autophagosome (green) to a lysosome (red). The mt-Keima protein is stable in lysosomes, and the excitation peak of mt-Keima shifts from 440 nm to 586 nm upon lysosome delivery, indicating mitophagy activation and delivery of mitochondria to lysosomes [25].

### 2.2. Preparation of Primary Neurons

Animal use was in accordance with local rules and with the regulations and ethical guidelines. Primary cortical neurons were prepared from embryonic day 14–16 pregnant C57BL/6 mice or mt-Keima mice as described previously [26]. Briefly, cortices from embryos in a single litter were dissected, meninges were removed, and tissue was pooled. Cortices were roughly chopped before incubation in 0.25% trypsin/EDTA followed by trituration. Cells were pelleted by centrifugation, resuspended, and plated in neurobasal medium supplemented with B27, 100 units/mL penicillin, 100 μg/mL streptomycin, 0.25 μg/mL amphotericin B, 300 μm glutamine and 25 μm 2-mercaptoethanol. Neurons were prepared and plated at a density of 30–50–70 × 10^3^ cells/well. Treatments were carried out on neurons grown for a minimum of 7 days in vitro, and all media were obtained from Invitrogen.

### 2.3. Sample Preparation for Live Cell Microscopy

Primary neurons cultured from mt-Keima mice were used for mitophagy and mitochondrial morphology analysis. Cells were washed with PBS and plated on Matek dishes of precision cover glasses thickness No. 1.5H (tol. ± 5 μm) in a 24-well plate, with 5 × 10^5^ cells per well, and left to adhere overnight at 37 °C in a cell culture incubator. Cells were washed gently three times with warm PBS. Further anti-bleaching live cell visualization medium (DMEMgfp-2, Evrogen, Moscow, Russia) was added to the cells 30 min before imaging. Images were acquired with a Zeiss LSM 880 Airyscan super-resolution system with live cell capabilities and fitted with a fast-AS module (Carl Zeiss, Oberkochen, Germany). Microscopes were equipped with an environmental chamber that maintained 37 °C with humidified 5% CO_2_ gas/95% air during imaging.

### 2.4. Oxygen-Glucose Deprivation (OGD) of Primary Neurons

Primary neurons were cultured for a minimum of 7 days (DIV7) from mt-Keima mice. Growth medium was replaced with de-gassed, glucose-free, neurobasal-A medium (Invitrogen, Life Technologies Glasgow, UK) and culture plates were maintained at 37 °C in a 95% N2/5% CO_2_ environment for the duration of the OGD. Following OGD, the medium was replaced with a standard neurobasal medium (containing additions described above) and cultures returned to 5% CO_2_/95% air incubation. For control plates, medium was replaced with standard Neurobasal media at the start of the OGD plates and replaced again with media after 90 min. Cells were collected at 0, 6 or 24 h following treatment.

### 2.5. Quantification of Mitophagy and Mitochondrial Morphology Analysis in Primary Neurons

Mitophagy was analyzed in control and OGD-induced cells in three independent experiments. Mitophagy in primary neurons was quantified as described before [24]. mt-Keima fluorescence signals from 561 nm laser excitation (acidic) in red and the signal from 458 nm laser excitation (neutral pH) in green were calculated. Mitophagy was calculated as the total number of red pixels divided by the total number of all pixels. For quantification of morphology, mitochondria were categorized based on length: fragmented (<1 µm), tubular (1–3 µm) and elongated (>3 µm), as described previously [27]. Over 20 cells were analyzed in control and exposed to OGD in three independent experiments. Volocity 6 (Perkin-Elmer, Waltham, MA, USA) and Imaris (version 8.4.2; Bitplane A.G., Zurich, Switzerland) were used for 3D rendering and to quantify mitochondrial length, volume, and number.

### 2.6. Measurement of Oxygen Consumption Rate (OCR)

Real-time measurements of oxygen consumption rates were performed on an XFe96 Seahorse extracellular flux analyzer (Seahorse Biosciences, North Billerica, MA, USA). The optimal seeding density and test compound concentrations were empirically determined before commencement of experiments. According to the methods described in the XFe96 extracellular flux analyzer user manual (Seahorse Bioscience), preliminary studies were run with Carbonyl cyanide-4-(trifluoromethoxy) phenylhydrazone (FCCP) to identify the optimal number of cells required to observe a sufficient shift in OCR. Once the cell number was decided, we determined the optimal working concentrations for each of the stimulating compounds used in the mitochondrial function analysis (oligomycin, FCCP, and rotenone). Cells were then plated into XFe96 cell culture plates (Seahorse Biosciences, North Billerica, MA, USA) at a density of 50,000/well in 80 µL of DMEM (Sigma–Aldrich, St. Louis, MO, USA). Cells were allowed to adhere overnight in a 37 °C incubator with 5% CO_2_.

The day prior to the experiment, 200 µL of XF calibration media was added to the XF sensor cartridges and kept in a non-CO_2_ incubator for 24 h. XF sensor cartridges were loaded with test compounds and OCR measured. OCR was measured by sequential injections of oligomycin (2 μM final concentration, blocks ATP synthase to assess respiration required for ATP turnover), FCCP (carbonyl cyanide 4-trifluoromethoxy-phenylhydrazone, 2 μM final concentration, a proton ionophore uncoupler inducing maximal respiration), and rotenone plus antimycin A (0.5 μM final concentration of each, which completely inhibits electron transport to measure non-mitochondrial respiration). Each step had three cycles; each cycle consisted of 3 min mixing, 2 min incubation and 3 min measurement. All experiments were run in three replicates with 3–4 samples per replicate. Cell counts were used to normalize OCR.

### 2.7. Hypoxia–Ischemia Procedure

At PND9, mice of both sexes were subjected to HI insult according to a method described previously with some modifications [28,29]. Briefly, mice were anesthetized with isoflurane (5.0% for induction and 1.5 to 3.0% for maintenance) in a 1:1 mixture of nitrogen and oxygen. The left common carotid artery was ligated, and the mice were returned to their cage and allowed to recover for one hour. The mice were then placed in an incubator perfused with a humidified gas mixture (10 ± 0.01% oxygen in nitrogen) at 36 °C for 70 min. After HI, the pups were returned to their dam until being sacrificed. The combination of artery ligation and hypoxia resulted in injury only in the hemisphere ipsilateral to the artery ligation (the left hemisphere), while no injury was produced in the contralateral hemisphere (the right hemisphere) [30].

### 2.8. Assessment of Mitophagy in the Brain Tissue of Mt-Keima Mice

Dual-excitation ratio imaging in brain tissues expressing mt-Keima was conducted with two sequential excitation lasers, 458 and 561 nm, in combination with a 570–695 nm emission range (for both excitation wavelengths). As the pH gradient across the lysosomal membrane was lost during fixation, all images needed to be obtained from freshly harvested brain tissue. All images were acquired within 1 h after dissection, following the original protocol [31]. The mt-Keima intensity with both excitations was found to be relatively stable within the first hour after dissection. The tissues were kept at 4 °C during this time. No more than two samples were analyzed in the same day. We imaged three regions adjacent to the injury at an interval 100 µm at 3 h, 8 h, 24 h and 7 days post HI using a Zeiss LSM 880 Airyscan super-resolution system.

### 2.9. Calculation of Mitophagy

Calculation of mitophagy based on mt-Keima signal was performed using Zeiss ZEN software on a pixel-by-pixel basis. Pixels were plotted in a scatter diagram based on their intensity level from each channel. Green intensity is shown on the x-axis, and red intensity is shown on the y-axis. As pixel intensity dictates its position on the scatterplot, pixels that have high red intensity are designated in the software using the crosshairs and quadrants. Pixels that have low-intensity levels in both channels are referred to as background and are not taken into consideration for analysis. The fold change of mitophagy was calculated by comparing in matched images the numbers of pixels that have high red intensity over the number of all pixels.

For quantitative analysis of mitophagy using mt-Keima, we defined the level of mitophagy as the total number of red pixels (Keima in acidic pH) divided by the total number of all pixels. The fold change of mitophagy was calculated by comparison of the number of pixels with high red intensity (quadrant 2) to the number of all pixels (quadrants (1 + 2 + 3 + 4) − background) in matched images that have the same experimental settings, including all imaging parameters used for data acquisition. In each experimental model, all imaging parameters remained the same for all data acquisition.

### 2.10. Quantitative Real-Time PCR

Brain tissue (*n* = 6 for all time points) after HI were used for total RNA extraction by using an miRNeasy mini kit (Qiagen, Solna, Sweden). Total RNA concentration was determined with a NanoDrop and the quality was checked by Experion RNA STDsense chip (Biorad). The RQI quality value was between 9 and 10 of all the RNA samples. Total RNA (1 μg) from each sample was used for first-strand cDNA synthesis according to the manufacturer’s instructions (QuantiTect reverse transcription kit, Qiagen). To determine mRNA expression, the cDNA samples were further processed by quantitative real-time (qRT)-PCR. Each PCR reaction (20 μL) contained 10 ng of cDNA, 10 μL of QuantiFast SYBR Green PCR master mix (Qiagen), 2 μL of PCR primers, and H_2_O to make a final reaction volume of 20 μL. A list of the PCR primers (Qiagen) used is shown in Supplementary Table S1.

### 2.11. Preparation of Protein Extracts, Electrophoresis, and Western Blotting

Total cell lysates were prepared from primary neuronal cultures. Brain tissue homogenates were prepared as previously described before [32]. Briefly, whole-brain tissue proteins were isolated by homogenization in RIPA buffer (Sigma Aldrich) added with complete Mini protease inhibitor cocktail tablets (Roche Diagnostics, Mannheim, Germany) and PhosSTOP phosphatase inhibitor cocktail tablets (Roche Diagnostics). The total protein concentration was quantified by BCA (Thermo Fisher Scientific, Rockford, IL, USA). The tissue extracts were boiled, equal amounts (25–30 μg) of the denatured protein per lane were loaded and separated on 26-well 4–20% TGX stain-free gels (Bio-Rad Laboratories GmbH, Munich, Germany). Total protein normalization was performed using Stain-free technology from Bio-Rad. Stain free blotting provides total protein loading controls in Western blots [32,33] ensuring both target and loading control are measured in a linear dynamic range. This technique uses a pre-cast gel containing an embedded proprietary trihalo compound from Bio-Rad, making proteins fluorescent directly using photoactivation. This allows the immediate visualization of proteins at any point on the gel or a membrane post-transfer without the need for additional stains such as Ponceau, Coomassie blue or Silver staining. After electrophoresis, the gel was placed into a stain-free enabled imager and the total protein signal was captured. Proteins were then transferred onto nitrocellulose membrane and the gel imaged post-transfer to ensure all protein was transferred successfully. Non-specific binding was blocked by incubation in 0.01 M Tris-buffered saline supplemented with 0.1% Tween 20 (TBST) containing 5% *w*/*v* non-fat dry milk for 1 h at room temperature. The membranes were then probed with different antibodies (Supplementary Table S2) in TBST containing 5% *w*/*v* non-fat dry milk, followed by an HRP conjugated secondary antibody. Signal was detected using the Super Signal West Dura Extended Duration Substrate (Thermo Fisher Scientific) and captured using a ChemiDoc MP Imaging System (Bio-Rad). Ultraviolet activation of the Criterion stain-free gel on a ChemiDoc MP Imaging System (Bio-Rad) was used to control for slight variations in protein loading. Band densitometry and quantification were performed using Image Laboratory (Version 5.0, Bio-Rad, Munich, Germany), and the protein band densities were normalized to the total-protein loading control in each sample without the need to use housekeeping proteins.

### 2.12. Statistical Analysis

Statistical analyses were conducted using GraphPad Prism version 9.00 for MAC (GraphPad Software, San Diego, CA, USA). Analyses included statistics compared by Kruskal–Wallis followed by Dunn’s multiple comparisons as well as Mann–Whitney U tests. A *p* value of <0.05 was considered statistically significant. Data are presented as mean ± SEM.

## 3. Results

### 3.1. Oxygen Glucose Deprivation (OGD) Alters Mitophagy and Induces Mitochondrial Fragmentation in Primary Neurons

**Figure 1:**
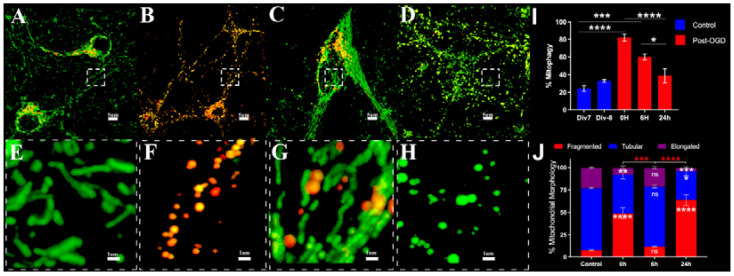
Changes in mitophagy and mitochondrial morphology in neurons following OGD by live cell airyscan imaging. (**A**)—Control, (**B**)—0 h post OGD, (**C**)—6 h post OGD, (**D**)—24 h post OGD, (**E**–**H**)—high power overlay. (**I**)—The graph represents changes in mitophagy over time. (**I**)—Div7 and Div 8 represent controls for 0, 6 h and 24 h post OGD. The data are for at least 14 cells per condition in three independent experiments. (**J**)—The graph shows the percentages of neurons with different forms of mitochondrial morphology in normoxic and OGD conditions * (inside stacked bars)—representing significant difference with respect to controls, * (outside stacked bars)—representing significant difference between time points * *p* ≤ 0.05, ** *p* ≤ 0.01; *** *p* ≤ 0.001; **** *p* ≤ 0.0001. Statistics compared by Kruskal–Wallis followed by Dunn’s multiple comparisons. The data are for at least 14 cells per condition in three independent experiments.

Primary neurons from mt-Keima mice were subjected to OGD for 90 min. Neurons under normoxic conditions and OGD were imaged live by super-resolution microscopy on a Carl Zeiss LSM 880 airyscan microscope. We noticed a significant increase in mitophagy at 0 h accompanied by an increase in ROS production (Supplementary Figure S1) and 6 h post-OGD. Simultaneous measurement of mitochondrial morphology showed an increase in fragmented (<3 μm) and decrease in elongated (>3 μm) mitochondria at 0 hand 24 h post-OGD with an in-between phase at 6 h where mitochondrial morphology normalized (Figure 1). Histogram plots of relative mitochondrial length demonstrated a shift to mitochondria with reduced length at 0 h, normalization at 6 h and a secondary phase of fragmentation at 24 h (Supplementary Figure S2A–D). The mean mitochondrial length was lower at 0 h (1.662 +/− 0.1003) but not at 6 h (2.779 +/− 0.015)) and 24 h (1.486 +/− 0.014) compared to controls (2.731 +/− 0.0213) (Supplementary Figure S2E).

### 3.2. OGD Reduces Mitochondrial Oxygen Consumption Rate in Primary Neurons

**Figure 2:**
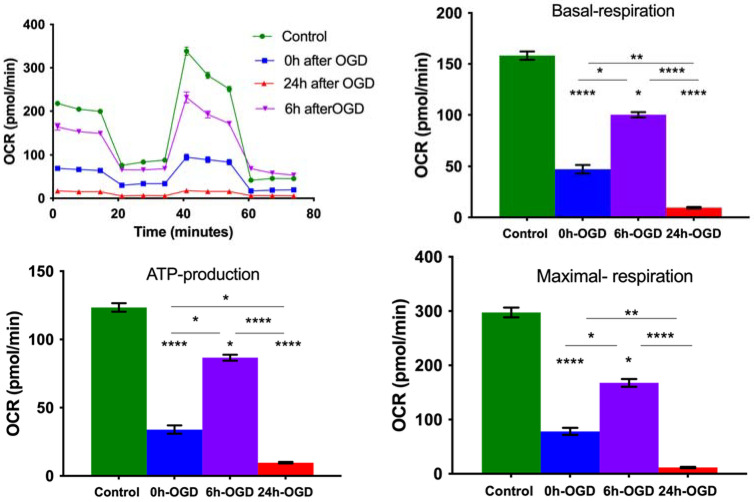
Measurement of mitochondrial respiration (OCR) and quantification of OCR. Bar graphs expressed as mean ± SEM calculating the difference between control, immediately after OGD and 24 h after OGD. * *p* ≤ 0.05, ** *p* ≤ 0.01; **** *p* ≤ 0.0001. Statistics compared by Kruskal–Wallis followed by Dunn’s multiple comparisons. The data are for Control 0 h-OGD 6 h-OGD 24 h-OGD for at least 20 wells per condition.

The mitochondrial oxygen consumption rate was measured by a seahorse XFe96 live cell metabolic analyzer with or without OGD for 90 min. OGD resulted in a decrease in basal respiration, maximal respiration, ATP-linked respiration and spare respiratory capacity (SRC) immediately after and 24 h post-OGD. A partial recovery of respiration occurred at 6 h after OGD indicating partial recovery of mitochondrial function followed by secondary energy failure (Figure 2).

### 3.3. Hypoxia–Ischemia Alters Mitophagy in the Neonatal Brain

**Figure 3:**
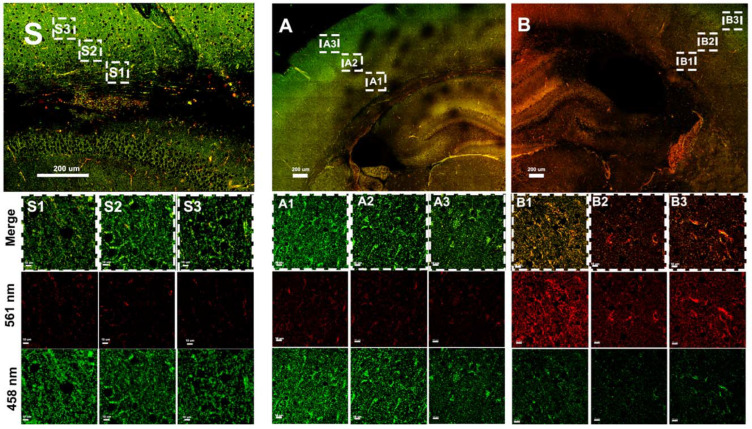
Assessment of mitophagy 3 h after HI in the brain tissue of mt-Keima mice by super-resolution microscopy; ((**A**), A1–A3) uninjured (contralateral) hemisphere; ((**B**), B1–B3) injured (ipsilateral) hemisphere; serial sections are visualized at 100 μm intervals (indicated in dashed rectangles) of both hemispheres and the in control ((**S**), S1–S3). The Keima protein changes color depending on the pH environment when being transferred from an autophagosome (green) to a lysosome (red). The mt-Keima protein is stable in lysosomes, and the excitation peak of mt-Keima shifts from 458 nm “green” to 561 nm “red” upon lysosome delivery, indicating mitophagy activation and delivery of mitochondria to lysosomes.

**Figure 4:**
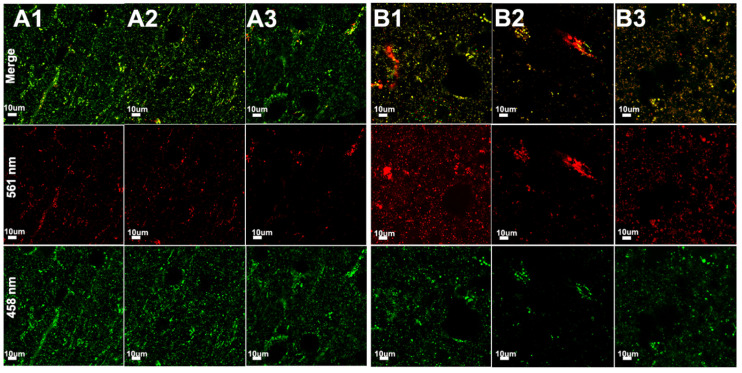
Assessment of mitophagy 8 h after HI in the brain tissue of mt-Keima mice by super resolution microscopy. (**A1**–**A3**): injured hemisphere (**B1**–**B3**) uninjured hemisphere; serial sections are visualized at 100 μm intervals of both hemispheres.

**Figure 5:**
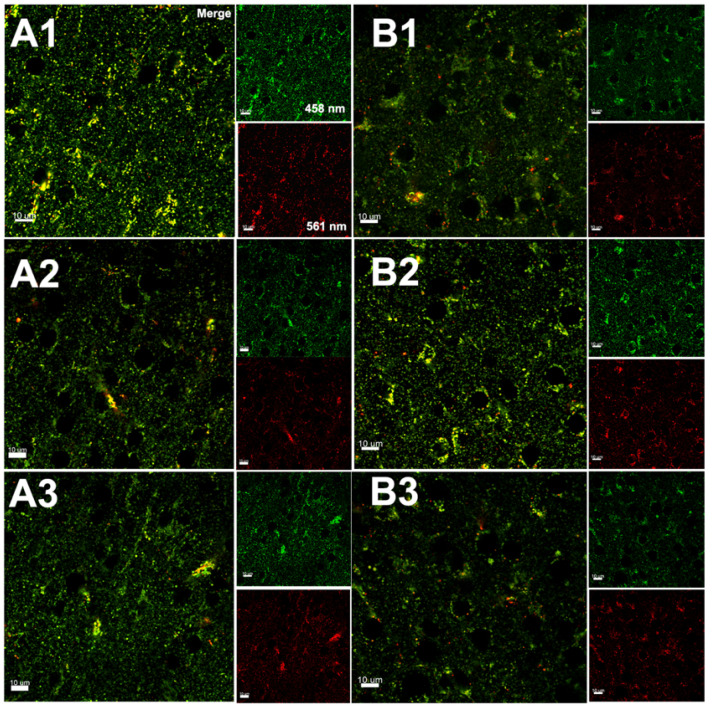
Assessment of mitophagy 24 h after HI in the brain tissue of mt-Keima mice by super-resolution microscopy. (**A1**–**A3**): Injured hemisphere, (**B1**–**B3**) uninjured hemisphere; serial sections are visualized at 100 μm intervals of both hemispheres. (**A1**–**B3**): Merge, red channel: excitation at 561 nm, green channel: excitation of 458 nm.

**Figure 6:**
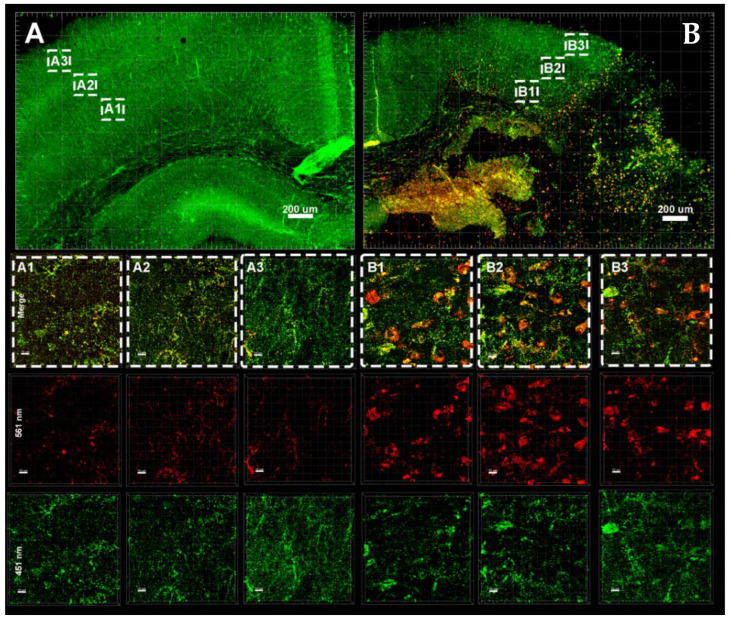
Assessment of mitophagy 72 h after HI in the brain tissue of mt-Keima mice by super-resolution microscopy. ((**A**), A1–A3): Injured hemisphere ((**B**), B1–B3), uninjured hemisphere; serial sections are visualized at 100 μm intervals (indicated as dashed rectangles) of both hemispheres.

**Figure 7:**
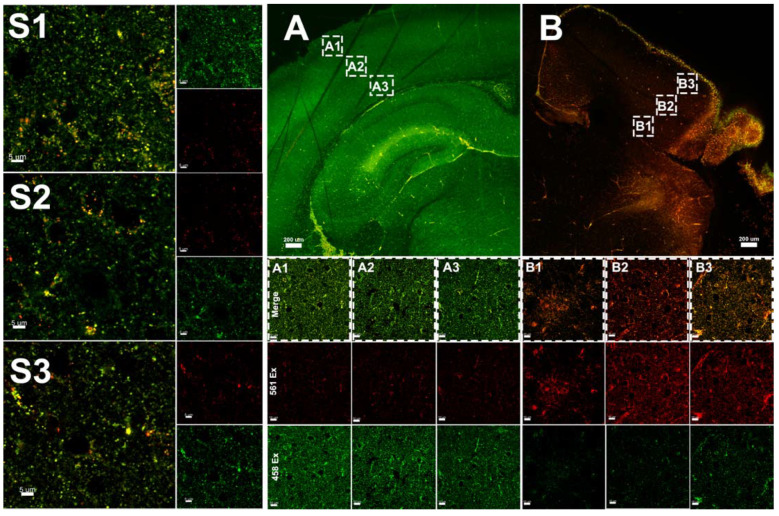
Assessment of mitophagy 7 days after HI in the brain tissue of mt-Keima mice by super-resolution microscopy; ((**A**), A1–A3) uninjured hemisphere; ((**B**), B1–B3) injured hemisphere; serial sections are visualized at 100 μm intervals (indicated in dashed rectangles) of both hemispheres. (A1–A3): injured hemisphere (B1–B3) uninjured hemisphere. (**S1**–**S3**) Control.

**Figure 8:**
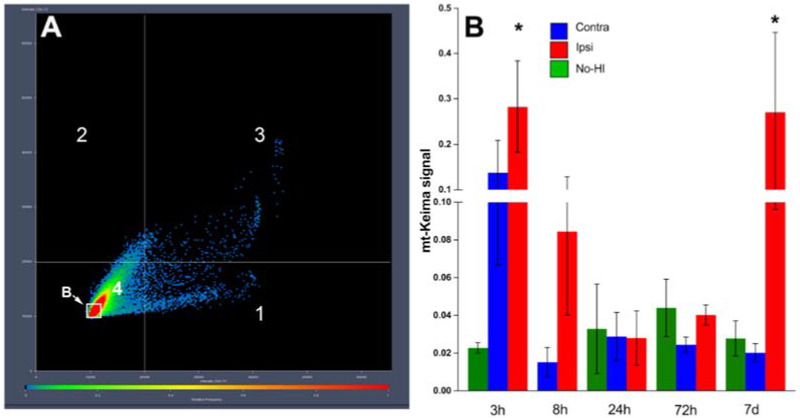
Assessment of mitophagy (**A**): mitophagy is quantified as the number of pixels in quadrant 1 divided by the total number of pixels. ((Quadrants 1 + 2 + 3 + 4) − B) where b is the background *n* = 3–6 mice/gp +/− SEM. * *p* ≤ 0.05. (**B**): Statistics compared by Mann–Whitney Test with respective No-HI controls.

On postnatal day 9, mt-Keima mice of both sexes were subjected to 70 min of HI. The insult results in injury in the ipsilateral (ipsi) hemisphere, typically in the cortex, hippocampus, and striatum, while there is no gross morphological injury in the contralateral (contra) hemisphere as previously reported by our group [30]. Following HI, mitophagy was assessed at 3 h (Figure 3), 8 h (Figure 4), 24 h (Figure 5), 72 h (Figure 6) and 7 days (Figure 7) in fresh brain tissue sampled in the border-zone of injury by super-resolution airyscan microscopy. The results show that mitophagy was transiently upregulated at 3 h after HI; thereafter, it was normalized at 24 h followed by a second wave of mitophagy at 7 days (Figure 8).

### 3.4. Hypoxia–Ischemia Upregulates Mitophagy Gene Expression

**Figure 9:**
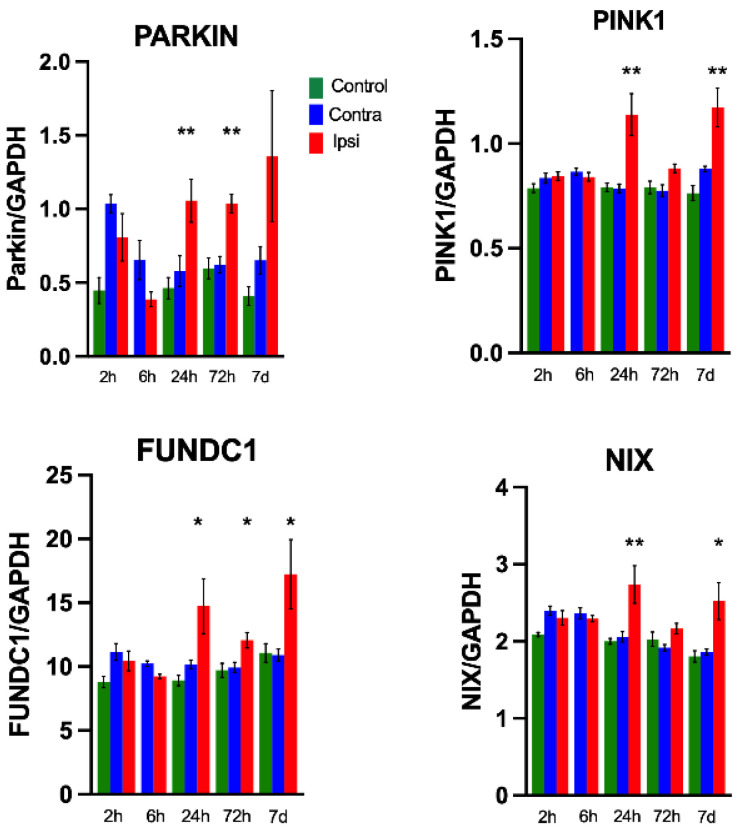
RT-PCR of mitophagy genes Pink1, Parkin, Nix, FUNDC1. N = 6 +/− SEM in duplicate. * *p* ≤ 0.05, ** *p* ≤ 0.01. Statistics compared by Mann–Whitney Test between the control and respective ipsilateral hemisphere.

The mRNA expression of PARKIN, PINK1, FUNDC1 and NIX genes was significantly upregulated at 24 h and 7 d after HI in the ipsilateral hemisphere compared to naïve controls animals. There was no change in the mRNA expression at other time points. No differences were found in the constitutive expression of the housekeeping gene GAPDH between HI and naïve animals (Figure 9).

### 3.5. Mitophagy Protein Expression Is Altered In Vitro in Primary Neurons after OGD and after HI in the Neonatal Brain

#### 3.5.1. Expression of Mitophagy-Related Proteins in Primary Neurons after OGD

**Figure 10:**
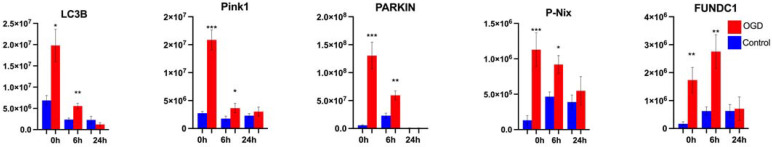
Quantification of mitophagy-related proteins after OGD in primary neurons, *n* = 6–8/gp +/− SEM. * *p* ≤ 0.05, ** *p* ≤ 0.01, *** *p* ≤ 0.001. Statistics compared by Mann–Whitney Test with respective controls.

The most well-characterized mechanism activating mitophagy relies on PTEN-induced putative kinase 1 (PINK1)/Parkin (Park et al., 2006). We measured protein levels of LC3B, PINK1/PARKIN, NIX and FUNDC1 using Western blots to investigate both PARKIN-dependent and -independent pathways. Most mitophagy proteins function as a binding partner for LC3B. In primary neurons, LC3B was upregulated immediately after and 6 h after OGD. Western blot analysis of PINK1/PARKIN1- and PINK1-independent proteins (NIX, p-NIX and FUNDC1) showed that both pathways were upregulated immediately after and 6 h after OGD (Figure 10). A total of 24 h after OGD there was a significant decrease in protein levels.

#### 3.5.2. Expression of Mitophagy-Related Proteins in Brain Homogenates after HI

**Figure 11:**
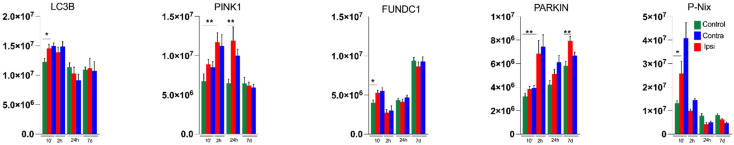
Quantification of mitophagy-related proteins after HI *n* = 6–8/gp +/− SEM. * *p* ≤ 0.05, ** *p* ≤ 0.01. Statistics compared by Mann–Whitney Test between the control and respective ipsilateral hemispheres.

In brain homogenates after HI in PND9 C57BL6J mice. Levels of LC3B, PINK1/PARKIN, NIX and FUNDC1 were quantified using Western blots to investigate both PARKIN-dependent and -independent pathways. The results show that LC3B was upregulated immediately (10 min) after HI. PINK1 was upregulated 2 h and 24 h after HI whereas PARKIN was upregulated 2 h and continued to be upregulated even 7 days after HI. FUNDC1 was upregulated at 10′ in the ipsilateral side which then decreases at 2 h after HI and normalized at 7 days after HI. Phosphorylated Nix (p-Nix) levels were also upregulated at 10 min in the ipsilateral side which then decreased at 2–3 h after HI and normalized at 7 days after HI (Figure 11).

## 4. Discussion

Dysregulation of mitochondrial energy metabolism is a characteristic hallmark feature in neurodegenerative disorders [34]. Mitochondria, at the center of energy metabolism, is a hub of injury responses [8]. Here, we investigate mitophagy in response to OGD in vitro and HI in vivo and alterations in mitophagy-related proteins. Mitochondrial dysfunction in neurons has a neurodegenerative component [35]. Post-mitotic neuronal cells are more inclined to mitochondrial impairment due to their increased energetic demands [36]. The balance of mitochondrial fission and fusion is important for adaptation to environmental situations and mitochondrial quality control [9]. Aged or dysfunctional mitochondria in cells need to be repaired through mitochondrial surveillance quality mechanisms eliminated via mitophagy. Any perturbation of mitochondrial dynamics (fission, fusion) and mitophagy can be deleterious. Therefore, it is critical to protect mitochondrial function especially in the hours following HI to safeguard neuronal survival, providing additional neuroprotection for infants where hypothermia alone is inadequate [21]. To this end it is important to understand mitophagy and its mechanisms, and thereby protect mitochondrial function as it represents a valid target for neuroprotective intervention after neonatal brain injury.

Mitophagy can be induced under certain physiological conditions as a stress-response mechanism [37] to eliminate selectively damaged mitochondria after depolarization of the mitochondrial membrane or in response to hypoxia [38]. Our results show that neurons under normoxic conditions primarily exhibited tubular mitochondria with a smaller percentage of fragmented or damaged mitochondria. OGD induced a significant increase in fragmented mitochondria and a decrease in elongated mitochondria. This was accompanied by an increase in mitophagy (Figure 1). We also found an increase in ROS production immediately after OGD (Supplementary Materials). Thereafter, mitophagy decreased from 6 to 24 h with normalization of mitochondrial morphology at 6 h which was accompanied by a recovery of mitochondrial respiration (Figure 2). At 24 h, we noticed an increase in mitochondrial fragmentation and decreases in tubular and elongated mitochondria accompanied by a reduction in mitochondrial respiration. This is primarily due to increased neuronal cell death (Supplementary Figure S3).

Following injury, the degree of mitochondrial fragmentation is reported to correlate with increases in mitophagy [18]. The increased population of fragmented mitochondria provides a better control of their integrity by facilitating the removal of damaged mitochondria through mitophagy [39]. Altered mitochondrial morphology provokes mitochondrial dysfunction, which can lead to an increase in the production of reactive oxygen species, and other adverse events, including disturbance of Ca^2+^ homeostasis [40]. Mitochondrial fragmentation is a well-documented response to deprivation of oxygen and glucose. For example, permanent middle cerebral artery occlusion (pMCAO) leads to fragmented mitochondria in adult rats [41]. Interestingly, biphasic mitochondrial fragmentation has previously been observed in vitro in response to OGD [42]. Here, we find that quantification of mitophagy in fresh brain tissue of mt-Keima mice revealed two distinct phases of mitophagy (Figure 8). A first phase occurs immediately after HI where we found an early increase in mitophagy. Thereafter, mitophagy normalized but was later followed by a delayed phase which occurred only in the injured ipsilateral hemisphere. We do not know at this point whether increased mitophagy aggravates or mitigates brain injury. Based on previous work [42], we hypothesize that early mitophagy contributes to secondary energy depletion, whereas secondary mitophagy could have beneficial effects on post-HI regeneration and repair. Our hypothesis aligns with the only other direct study of mitophagy in the neonatal brain [18], where the authors investigated sex-dependent mitophagy and neuronal death following HI in postnatal day 7 rats. Evaluation of mitophagy at 24 h post HI identified increased mitophagy in female rat brain compared with male rat brain and subsequently less injury [18], which may suggest that mitophagy induced at this later time point contributes to neuroprotection.

Mitophagy follows a common pattern involving a receptor-mediated mechanism whereby the receptors physically attach the mitochondria to be recycled with LC3B, the principal component of the autophagosomal membrane via the LC3-interacting region (LIR) [43]. The nature and origin of mitophagy receptors can change according to the type of mitophagy, based on whether receptors are localized in the mitochondrial membrane, or if they are non-mitochondrial proteins that identify and bind ubiquitinated chains on the mitochondrial surface and LC3B on the nascent autophagosome structure [44]. Here, we investigated mitophagy mediated directly by mitochondrial membrane receptors which includes NIX and FUNDC1. We also investigated non-mitochondrial receptor-mediated or PINK1/Parkin-mediated mitophagy at 0 h (immediately after) 6 h and 24 h post OGD (Figure 10). As expected, we noticed an increase in LC3B, which was most pronounced immediately after OGD. We also found that P-NIX, FUNDC1, PINK1 and Parkin increased at 0 h and 6 h but not at 24 h, which is probably due to loss of neuronal viability accompanied by loss of oxygen consumption rate (OCR) (Supplementary Figure S3, Figure 2).

Mammalian cells harbor multiple mitophagy pathways that can be triggered in response to diverse stimuli [45]. NIX participates in mitochondrial priming by promoting Parkin translocation to the mitochondria [46,47]. Nix can also serve as an alternative mediator of mitophagy in the absence of PINK1/Parkin-mediated mitophagy [48]. Ruo et al. have demonstrated that silencing NIX using knockout mice conferred significant neuroprotection and rescues neurons from HI-induced apoptosis and excessive mitophagy both in vivo and in vitro [22]. These results, together with other studies [49], indicate that early mitophagy associates with NIX ablation are deleterious and aggravate brain injury. Quantification of proteins was performed immediately after HI injury (at 10 min), as well as 2 h and 7 days post HI. NIX and FUNDC1 bind directly to LC3B to target damaged mitochondria to autophagosomes [50]. We found a significant increase in phosphorylated NIX levels 10 min after HI. Phosphorylation of Nix enhances its interaction with LC3 proteins and subsequently induces receptor-mediated mitophagy [51,52]. FUNDC1 functions as a mitophagy receptor and plays an important role in hypoxia-induced mitophagy. It has been suggested that, under hypoxic conditions, the MARCH5/FUNDC1 axis is involved in sensing the stress signal and fine-tuning the mitophagy response [53]. The degradation of mitochondrial proteins is prevented to a remarkable extent by knockdown of FUNDC1 [50], supporting the notion that early induction of excessive mitophagy might be deleterious. Our data show that there is an increase in FUNDC1 levels immediately after HI in the ipsilateral and contralateral hemisphere which is only exposed to hypoxia.

PINK1/Parkin is the most well investigated pathway of mitophagy. PINK1 functions upstream of Parkin in a common pathway [54]. PINK1 overexpression increases mitochondrial fragmentation, whereas its knockdown has opposite effects in rat hippocampal neurons [55]. We observed a robust activation of the PINK1 immediately after and 24 h after HI. Selective accumulation of PINK1 on the damaged mitochondria recruits Parkin, which, in turn, induces the degradation of the damaged mitochondria [56]. However, PINK1 is not just an initiator for recruiting Parkin, but is a central regulator for inducing mitophagy, and Parkin serves as an amplifier to generate more ubiquitin substrate for PINK1 to phosphorylate, inducing robust and rapid mitophagy induction [57]. PINK1-generated phospho-ubiquitin serves as the autophagy signal on mitochondria, and parkin then continues to act to amplify this signal [58]. Enzymatic impairment of Parkin has been proposed to produce toxic accumulation of its substrate proteins, which in turn causes neurodegeneration [59]. It is shown that the downregulation of PINK1 and Parkin impede the activation of the PINK1/Parkin pathway, inhibiting mitophagy, and consequently aggravates neuronal damage at the late phase of reperfusion after transient global cerebral ischemia [60]. We also found a time-dependent increase in PARKIN at 7 days, implicating the importance of this pathway in the different phases of mitophagy after HI. Taken together, this evidence suggests that late mitophagy may be protective in the neonatal brain.

## 5. Conclusions

**Figure 12:**
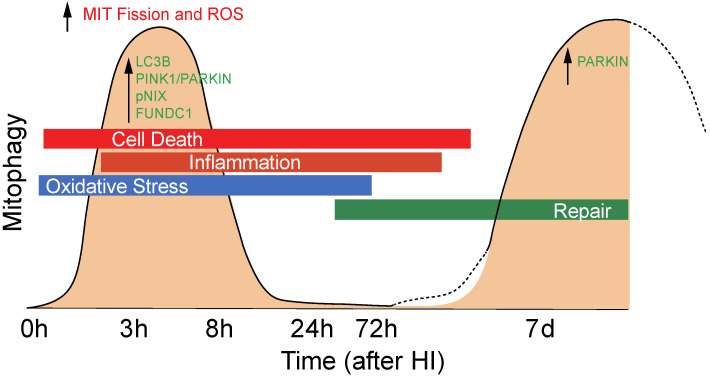
Hypothetical scheme for mitophagy. Following HI, mitophagy was transiently upregulated at 3 h after HI followed by a second wave at 7 days after HI. Western blotting results suggest both PINK1/Parkin-dependent and -independent mechanisms, including NIX and FUNDC1 are up-regulated immediately after HI, whereas Parkin-mediated mitophagy predominated 7 days after neonatal HI.

In primary neurons, we found a primary fission wave immediately after OGD with a significant increase in mitophagy. In vivo, following HI, mitophagy was upregulated at 3 h and 7 days after HI. Western blotting results suggest both PINK1/Parkin-dependent and -independent mechanisms, including NIX and FUNDC1, were upregulated immediately after HI, whereas increase in Parkin predominated at 7 days after neonatal HI. The role of mitophagy for the recovery after HI is unknown. From the current data, we speculate early mitophagy could lead to a substantial loss of mitochondria and result in secondary energy failure, whereas secondary mitophagy may be part of regeneration and repair mostly mediated by Parkin-dependent mechanisms. (Figure 12).

## Data Availability

The data presented in this study are available on request from the corresponding author.

## Supplementary Materials

The following supporting information can be downloaded at: https://www.mdpi.com/article/10.3390/cells11071193/s1, Supplementary Table S1: list of the PCR primers (Qiagen) used, Supplementary Table S2: List of antibodies used for Western blotting, Supplementary Figure S1: Mitosox fluorescence by live cell airyscan microscopy, Supplementary Figure S2: Histogram plots of relative distributions of mitochondrial lengths, Supplementary Figure S3: Cell viability after OGD.

## References

[1] Blomgren K., Hagberg H. (2006). Free radicals, mitochondria, and hypoxia-ischemia in the developing brain. Free Radic. Biol. Med..

[2] Niatsetskaya Z.V., Sosunov S.A., Matsiukevich D., Utkina-Sosunova I.V., Ratner V.I., Starkov A.A., Ten V.S. (2012). The Oxygen Free Radicals Originating from Mitochondrial Complex I Contribute to Oxidative Brain Injury Following Hypoxia-Ischemia in Neonatal Mice. J. Neurosci..

[3] Ten V.S., Starkov A. (2012). Hypoxic-Ischemic Injury in the Developing Brain: The Role of Reactive Oxygen Species Originating in Mitochondria. Neurol. Res. Int..

[4] Youle R.J., Narendra D.P. (2011). Mechanisms of mitophagy. Nat. Rev. Mol. Cell Biol..

[5] Bingol B., Sheng M. (2016). Mechanisms of mitophagy: PINK1, Parkin, USP30 and beyond. Free Radic. Biol. Med..

[6] Puka-Sundvall M., Gajkowska B., Cholewinski M., Blomgren K., Lazarewicz J.W., Hagberg H. (2000). Subcellular distribution of calcium and ultrastructural changes after cerebral hypoxia-ischemia in immature rats. Dev. Brain Res..

[7] Northington F., Zelaya M., O’Riordan D., Blomgren K., Flock D., Hagberg H., Ferriero D., Martin L. (2007). Failure to complete apoptosis following neonatal hypoxia–ischemia manifests as “continuum” phenotype of cell death and occurs with multiple manifestations of mitochondrial dysfunction in rodent forebrain. Neuroscience.

[8] Hagberg H., Mallard C., Rousset C.I., Thornton C. (2014). Mitochondria: Hub of injury responses in the developing brain. Lancet Neurol..

[9] Baburamani A.A., Hurling C., Stolp H., Sobotka K., Gressens P., Hagberg H., Thornton C. (2015). Mitochondrial Optic Atrophy (OPA) 1 Processing Is Altered in Response to Neonatal Hypoxic-Ischemic Brain Injury. Int. J. Mol. Sci..

[10] Swerdlow N., Wilkins H. (2020). Mitophagy and the Brain. Int. J. Mol. Sci..

[11] Doxaki C., Palikaras K. (2020). Neuronal Mitophagy: Friend or Foe?. Front. Cell Dev. Biol..

[12] De Oliveira L.G., Angelo Y.D.S., Iglesias A.H., Peron J.P.S. (2021). Unraveling the Link Between Mitochondrial Dynamics and Neuroinflammation. Front. Immunol..

[13] Giorgi C., Bouhamida E., Danese A., Previati M., Pinton P., Patergnani S. (2021). Relevance of Autophagy and Mitophagy Dynamics and Markers in Neurodegenerative Diseases. Biomedicines.

[14] Lemasters J.J., Nieminen A.-L., Qian T., Trost L.C., Elmore S.P., Nishimura Y., Crowe R.A., Cascio W.E., Bradham C.A., Brenner D.A. (1998). The mitochondrial permeability transition in cell death: A common mechanism in necrosis, apoptosis and autophagy. Biochim. Biophys. Acta.

[15] Leaw B., Nair S., Lim R., Thornton C., Mallard C., Hagberg H. (2017). Mitochondria, Bioenergetics and Excitotoxicity: New Therapeutic Targets in Perinatal Brain Injury. Front. Cell. Neurosci..

[16] Hoffmann A., Spengler D. (2018). The Mitochondrion as Potential Interface in Early-Life Stress Brain Programming. Front. Behav. Neurosci..

[17] Ivankovic D., Chau K., Schapira A., Gegg M.E. (2016). Mitochondrial and lysosomal biogenesis are activated following PINK 1/parkin-mediated mitophagy. J. Neurochem..

[18] Demarest T., Waite E., Kristian T., Puche A., Waddell J., McKenna M., Fiskum G. (2016). Sex-dependent mitophagy and neuronal death following rat neonatal hypoxia–ischemia. Neuroscience.

[19] McWilliams T.G., Muqit M.M. (2017). PINK1 and Parkin: Emerging themes in mitochondrial homeostasis. Curr. Opin. Cell Biol..

[20] Wu X., Zheng Y., Liu M., Li Y., Ma S., Tang W., Yan W., Cao M., Zheng W., Jiang L. (2020). BNIP3L/NIX degradation leads to mitophagy deficiency in ischemic brains. Autophagy.

[21] Thornton C., Jones A., Nair S., Aabdien A., Mallard C., Hagberg H. (2018). Mitochondrial dynamics, mitophagy and biogenesis in neonatal hypoxic-ischaemic brain injury. FEBS Lett..

[22] Shi R.-Y., Zhu S.-H., Li V., Gibson S., Xu X., Kong J.-M. (2014). BNIP3 Interacting with LC3 Triggers Excessive Mitophagy in Delayed Neuronal Death in Stroke. CNS Neurosci. Ther..

[23] Kabeya Y., Mizushima N., Ueno T., Yamamoto A., Kirisako T., Noda T., Kominami E., Ohsumi Y., Yoshimori T. (2000). LC3, a mammalian homologue of yeast Apg8p, is localized in autophagosome membranes after processing. EMBO J..

[24] Sun N., Yun J., Liu J., Malide D., Liu C., Rovira I.I., Holmström K., Fergusson M.M., Yoo Y.H., Combs C.A. (2015). Measuring In Vivo Mitophagy. Mol. Cell.

[25] Katayama H., Kogure T., Mizushima N., Yoshimori T., Miyawaki A. (2011). A Sensitive and Quantitative Technique for Detecting Autophagic Events Based on Lysosomal Delivery. Chem. Biol..

[26] Thornton C., Bright N.J., Sastre M., Muckett P.J., Carling D. (2011). AMP-activated protein kinase (AMPK) is a tau kinase, activated in response to amyloid β-peptide exposure. Biochem. J..

[27] Nair S., Sobotka K.S., Joshi P., Gressens P., Fleiss B., Thornton C., Mallard C., Hagberg H. (2019). Lipopolysaccharide-induced alteration of mitochondrial morphology induces a metabolic shift in microglia modulating the inflammatory response in vitro and in vivo. Glia.

[28] Rice J.E., Vannucci R.C., Brierley J.B. (1981). The influence of immaturity on hypoxic-ischemic brain damage in the rat. Ann. Neurol..

[29] Hedtjärn M., Leverin A.-L., Eriksson K., Blomgren K., Mallard C., Hagberg H. (2002). Interleukin-18 Involvement in Hypoxic–Ischemic Brain Injury. J. Neurosci..

[30] Svedin P., Hagberg H., Sävman K., Zhu C., Mallard C. (2007). Matrix Metalloproteinase-9 Gene Knock-out Protects the Immature Brain after Cerebral Hypoxia–Ischemia. J. Neurosci..

[31] Sun N., Malide D., Liu J., Rovira I.I., Combs C.A., Finkel T. (2017). A fluorescence-based imaging method to measure in vitro and in vivo mitophagy using mt-Keima. Nat. Protoc..

[32] Colella A.D., Chegenii N., Tea M.N., Gibbins I.L., Williams K.A., Chataway T.K. (2012). Comparison of Stain-Free gels with traditional immunoblot loading control methodology. Anal. Biochem..

[33] Gürtler A., Kunz N., Gomolka M., Hornhardt S., Friedl A.A., McDonald K., Kohn J.E., Posch A. (2013). Stain-Free technology as a normalization tool in Western blot analysis. Anal. Biochem..

[34] Rona-Voros K., Weydt P. (2010). The Role of PGC-1α in the Pathogenesis of Neurodegenerative Disorders. Curr. Drug Targets.

[35] Motori E., Atanassov I., Kochan S.M.V., Folz-Donahue K., Sakthivelu V., Giavalisco P., Toni N., Puyal J., Larsson N.-G. (2020). Neuronal metabolic rewiring promotes resilience to neurodegeneration caused by mitochondrial dysfunction. Sci. Adv..

[36] Chen H., Chan D.C. (2009). Mitochondrial dynamics-fusion, fission, movement, and mitophagy-in neurodegenerative diseases. Hum. Mol. Genet..

[37] Frank M., Duvezin-Caubet S., Koob S., Occhipinti A., Jagasia R., Petcherski A., Ruonala M.O., Priault M., Salin B., Reichert A.S. (2012). Mitophagy is triggered by mild oxidative stress in a mitochondrial fission dependent manner. Biochim. Biophys. Acta.

[38] Song Y., Du Y., Zou W., Luo Y., Zhang X., Fu J. (2018). Involvement of impaired autophagy and mitophagy in Neuro-2a cell damage under hypoxic and/or high-glucose conditions. Sci. Rep..

[39] Mironova G.D., Pavlik L.L., Kirova Y.I., Belosludtseva N.V., Mosentsov A.A., Khmil N.V., Germanova E.L., Lukyanova L.D. (2019). Effect of hypoxia on mitochondrial enzymes and ultrastructure in the brain cortex of rats with different tolerance to oxygen shortage. J. Bioenerg. Biomembr..

[40] Belosludtsev K.N., Dubinin M.V., Talanov E.Y., Starinets V.S., Tenkov K.S., Zakharova N.M., Belosludtseva N.V. (2020). Transport of Ca^2+^ and Ca^2+^-Dependent Permeability Transition in the Liver and Heart Mitochondria of Rats with Different Tolerance to Acute Hypoxia. Biomolecules.

[41] Zuo W., Zhang S., Xia C.-Y., Guo X.-F., He W.-B., Chen N.-H. (2014). Mitochondria autophagy is induced after hypoxic/ischemic stress in a Drp1 dependent manner: The role of inhibition of Drp1 in ischemic brain damage. Neuropharmacology.

[42] Kumar R., Bukowski M.J., Wider J.M., Reynolds C., Calo L., Lepore B., Tousignant R., Jones M., Przyklenk K., Sanderson T.H. (2016). Mitochondrial dynamics following global cerebral ischemia. Mol. Cell. Neurosci..

[43] Wild P., McEwan D.G., Dikic I. (2014). The LC3 interactome at a glance. J. Cell Sci..

[44] Rodolfo C., Campello S., Cecconi F. (2018). Mitophagy in neurodegenerative diseases. Neurochem. Int..

[45] Wei H., Liu L., Chen Q. (2015). Selective removal of mitochondria via mitophagy: Distinct pathways for different mitochondrial stresses. Biochim. Biophys. Acta Bioenerg..

[46] Ding W.-X., Ni H.-M., Li M., Liao Y., Chen X., Stolz D.B., Dorn G.W., Yin X.-M. (2010). Nix Is Critical to Two Distinct Phases of Mitophagy, Reactive Oxygen Species-mediated Autophagy Induction and Parkin-Ubiquitin-p62-mediated Mitochondrial Priming. J. Biol. Chem..

[47] Wang K., Klionsky D.J., Hayat M.A. (2014). Molecular process and physiological significance of mitophagy. Autophagy: Cancer, Other Pathologies, Inflammation, Immunity, Infection, Aging.

[48] Koentjoro B., Park J.-S., Sue C.M. (2017). Nix restores mitophagy and mitochondrial function to protect against PINK1/Parkin-related Parkinson’s disease. Sci. Rep..

[49] Zhang J., Yuan G., Liang T., Pan P., Li X., Li H., Shen H., Wang Z., Chen G. (2020). Nix Plays a Neuroprotective Role in Early Brain Injury After Experimental Subarachnoid Hemorrhage in Rats. Front. Neurosci..

[50] Liu L., Feng D., Chen G., Chen M., Zheng Q., Song P., Ma Q., Zhu C., Wang R., Qi W. (2012). Mitochondrial outer-membrane protein FUNDC1 mediates hypoxia-induced mitophagy in mammalian cells. Nat. Cell Biol..

[51] Rogov V.V., Suzuki H., Marinković M., Lang V., Kato R., Kawasaki M., Buljubasic M., Šprung M., Rogova N., Wakatsuki S. (2017). Phosphorylation of the mitochondrial autophagy receptor Nix enhances its interaction with LC3 proteins. Sci. Rep..

[52] Marinković M., Šprung M., Novak I. (2021). Dimerization of mitophagy receptor BNIP3L/NIX is essential for recruitment of autophagic machinery. Autophagy.

[53] Chen Z., Liu L., Cheng Q., Li Y., Wu H., Zhang W., Wang Y., Sehgal S.A., Siraj S., Wang X. (2017). Mitochondrial E3 ligase MARCH 5 regulates FUNDC 1 to fine-tune hypoxic mitophagy. EMBO Rep..

[54] Vincow E.S., Merrihew G., Thomas R.E., Shulman N.J., Beyer R.P., MacCoss M.J., Pallanck L.J. (2013). The PINK1–Parkin pathway promotes both mitophagy and selective respiratory chain turnover in vivo. Proc. Natl. Acad. Sci. USA.

[55] Yu W., Sun Y., Guo S., Lu B. (2011). The PINK1/Parkin pathway regulates mitochondrial dynamics and function in mammalian hippocampal and dopaminergic neurons. Hum. Mol. Genet..

[56] Narendra D.P., Jin S.M., Tanaka A., Suen D.-F., Gautier C.A., Shen J., Cookson M.R., Youle R.J. (2010). PINK1 Is Selectively Stabilized on Impaired Mitochondria to Activate Parkin. PLoS Biol..

[57] Lazarou M. (2015). Keeping the immune system in check: A role for mitophagy. Immunol. Cell Biol..

[58] Lazarou M., Sliter D.A., Kane L.A., Sarraf S.A., Wang C., Burman J.L., Sideris D.P., Fogel A.I., Youle R.J. (2015). The ubiquitin kinase PINK1 recruits autophagy receptors to induce mitophagy. Nature.

[59] Romaní-Aumedes J., Canal M., Martín-Flores N., Sun X., Perez-Fernandez V., Wewering S., Fernández-Santiago R., Ezquerra M., Pont-Sunyer C., Lafuente A. (2014). Parkin loss of function contributes to RTP801 elevation and neurodegeneration in Parkinson’s disease. Cell Death Dis..

[60] Wen H., Li L., Zhan L., Zuo Y., Li K., Qiu M., Li H., Sun W., Xu E. (2021). Hypoxic postconditioning promotes mitophagy against transient global cerebral ischemia via PINK1/Parkin-induced mitochondrial ubiquitination in adult rats. Cell Death Dis..

