# Supplementary material for: Induction of Mitochondrial Fragmentation and Mitophagy after Neonatal Hypoxia–Ischemia
Source: Cells. 2022 Apr 1;11(7):1193. doi: 10.3390/cells11071193 (PMC8997592; doi:10.3390/cells11071193)
Supplement: Supplementary file 1 [file cells-11-01193-s001.zip › cells-1541658-supplementary.pdf]

Table S1: list of the PCR primers (Qiagen) used

Gene	Target protein and abbreviation	Reference
Pink1	PTEN induced putative kinase 1	QT00111349
OPTN	OPTINEURIN	QT00134078
Parkin	E3 ubiquitin-protein ligase parkin	QT00168462
BNIP3	BCL2/adenovirus E1B 19 kDa protein-interacting protein 3	QT02520126
Nix	BCL2/adenovirus E1B interacting protein 3-like	QT0012095
FUNDC1	FUN14 domain containing 1	QT01047123
Pgam5	Phosphoglycerate mutase family member 5	QT01073877
MUL1	Mitochondrial ubiquitin ligase activator of NFKB 1	QT00132734
GAPDH	glyceraldehyde-3-phosphate dehydrogenase	QT01658692

Table S2: List of antibodies used for western blotting

Protein	Antibody used	Dilution	Size
P-nix	ab208190, Abcam	1:1000	37kDa
FUNDC1	ab224722, Abcam	1:2000	18kDa
LC3b	#2775, Cell Signaling	1:750	14-16kDa
Parkin	sc-32282, SantaCruz Bio.	1:200	52kDa
Pink-1	BC100-494, Novus Bio.	1:3000	63kDa

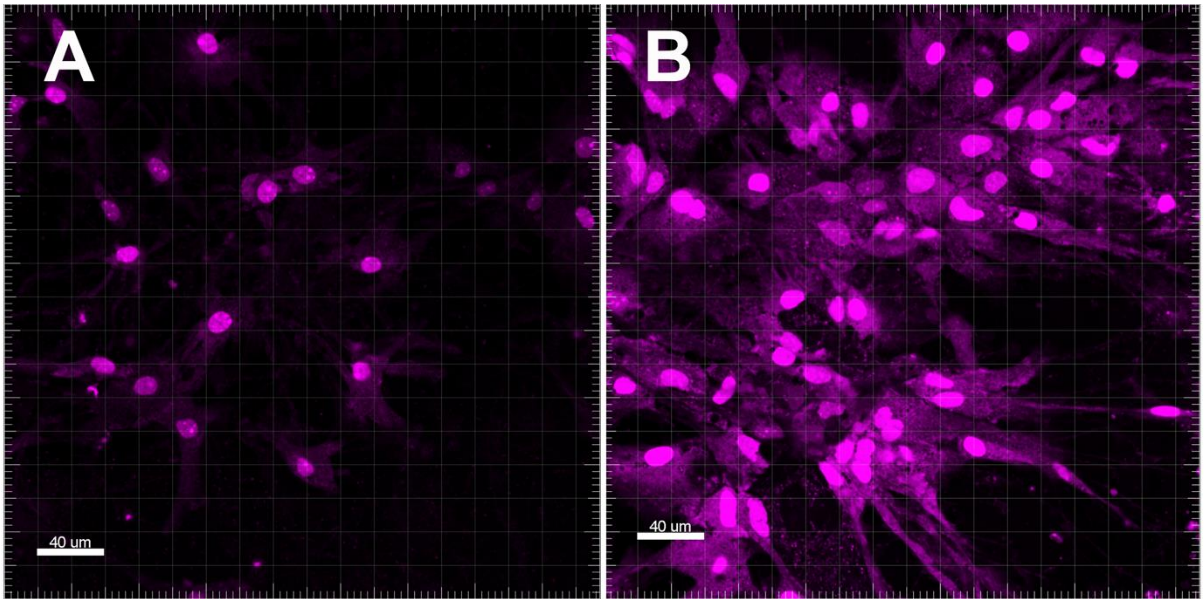

Figure S1: Mitosox fluorescence by live cell airyscan microscopy (A) Control (B) Immediately after OGD.

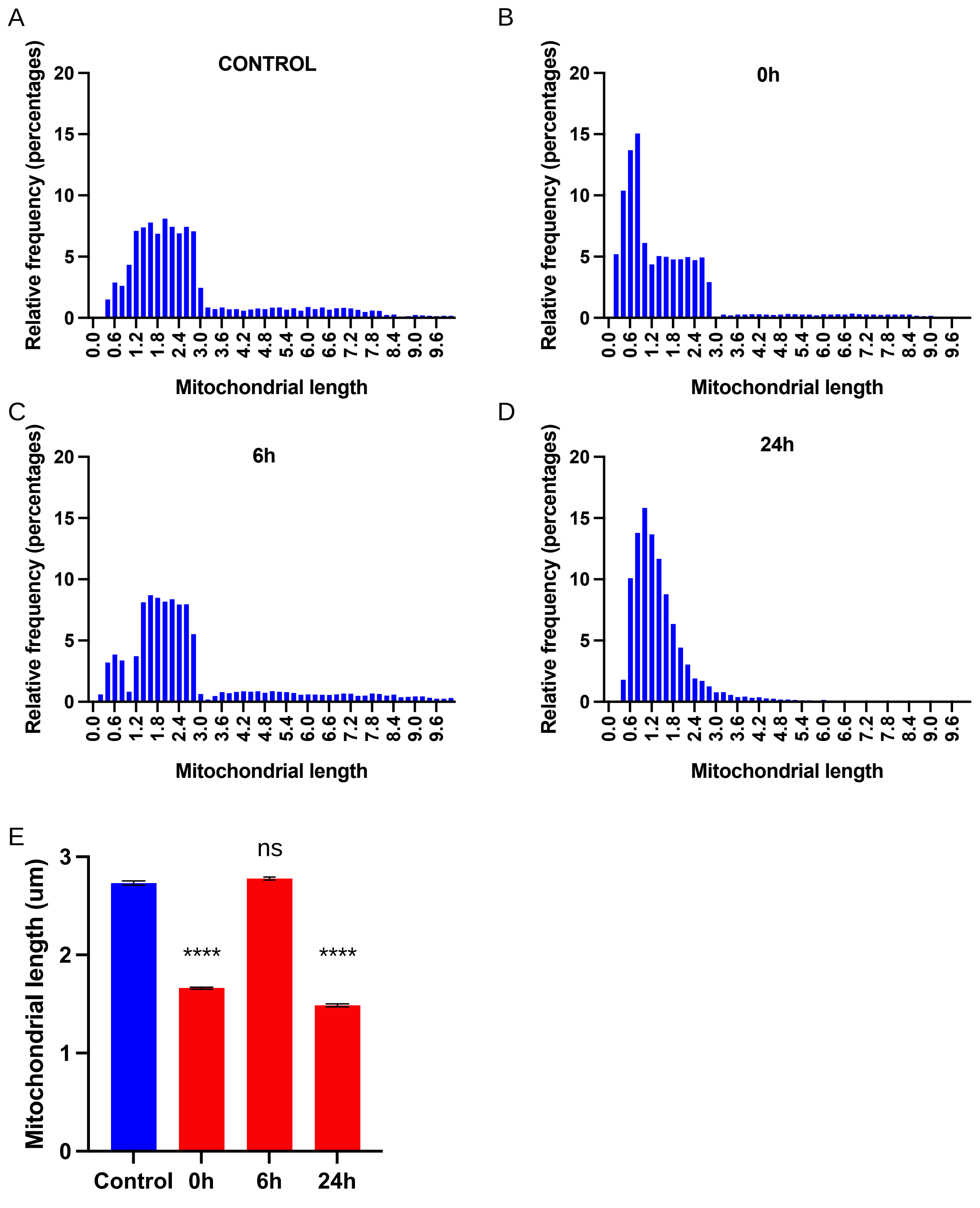

Supplementary Figure S2: Relative distributions of mitochondrial lengths.Histogram plots show the relative distributions of mitochondrial length for Control (A), 0h (B), 6h (C), and 24h (D) after OGD. (E): Average mitochondrial length at these time points. Statistics compared by Kruskal-Wallis followed by Dunn's multiple comparisons. The data are for at least 14 cells per condition in three independent experiments.

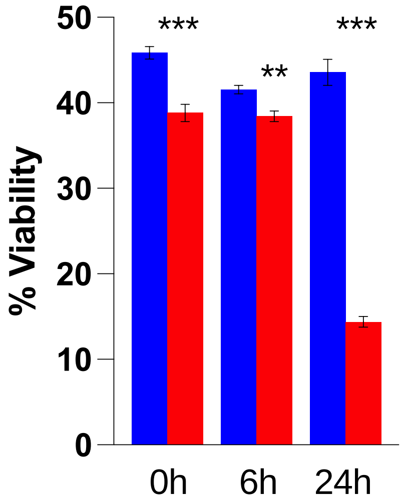

Supplementary Figure S3: Cell viability after OGD determined using a NucleoCounter® NC-200™ (Chemometec, Denmark). Percentage viability was determined using the formula below The data are for n= 5-9 wells per condition in three independent experiments. Statistics compared by Mann-Whitney Test between OGD (red bars) with respective controls (blue bars)

$$\%\,Viability = \frac{total\ number\ of\ cells/ml\ - number\ of\ nonviable\ cells/ml}{total\ number\ of\ cells/ml} \times 100$$
